# Genistein attenuated oxidative stress, inflammation, and apoptosis in L-arginine induced acute pancreatitis in mice

**DOI:** 10.1186/s12906-022-03689-9

**Published:** 2022-08-04

**Authors:** Prasong Siriviriyakul, Jumlongluk Sriko, Kanjana Somanawat, Maneerat Chayanupatkul, Naruemon Klaikeaw, Duangporn Werawatganon

**Affiliations:** 1 Alternative and Complementary Medicine for Gastrointestinal and Liver Diseases Research Unit, Department of Physiology, Faculty of Medicine, Chulalongkorn University, 1873 Rama 4 Road, Pathumwan, Bangkok, 10330 Thailand; 2 Department of Pathology, Faculty of Medicine, Chulalongkorn University, Bangkok, 10330 Thailand

**Keywords:** Acute pancreatitis, Anti-apoptosis, Anti-inflammation, Anti-oxidant, Genistein, Mice

## Abstract

**Aim:**

Acute pancreatitis is a common and potentially serious condition. However, a specific treatment for this condition is still lacking. Genistein, with its anti-oxidant and anti-inflammatory effects, could possibly be used to tackle the underlying pathophysiology of acute pancreatitis. Therefore, the aim of this study was to investigate the effects of genistein on oxidative stress, inflammation, and apoptosis in acute pancreatitis induced by L-arginine in mice.

**Methods:**

Twenty-four male ICR mice were equally divided into 4 groups: Control (Con); Acute pancreatitis (AP) group: Two doses of i.p. 350 mg/100 g body weight (BW) of L-arginine were administered 1 h apart; AP and low-dose genistein (LG) group: mice were given i.p. injection of 10 mg/kg genistein 2 h prior to L-arginine injection followed by once-daily dosing for 3 days; and AP and high-dose genistein (HG) group: mice were given 100 mg/kg genistein with the similar protocol as the LG group. Pancreatic tissue was evaluated for histopathological changes and acinar cell apoptosis, malondialdehyde (MDA) levels, immunohistochemical staining for myeloperoxidase (MPO), nuclear factor-kappa beta (NF-kB), and 4-hydroxynonenal (4-HNE). Serum levels of amylase (AMY), c-reactive protein (CRP), and interleukin (IL)-6 were measured.

**Results:**

Significant increases in the degree of acinar cell apoptosis, pancreatic MDA, serum IL-6 and amylase, MPO, NF-kB and 4-HNE positivity were observed in the AP group. All these parameters declined after low- and high-dose genistein treatment. Severe pancreatic inflammation, edema, and acinar cell necrosis were observed in the AP group. Significant improvement of histopathological changes was seen in both low- and high-dose genistein groups. There were no significant differences in any parameters between low and high doses of genistein.

**Conclusion:**

Genistein could attenuate the severity of histopathological changes in acute pancreatitis through its anti-oxidant, anti-inflammatory, and anti-apoptotic properties.

**Supplementary Information:**

The online version contains supplementary material available at 10.1186/s12906-022-03689-9.

## Introduction

Acute pancreatitis (AP) is an acute inflammatory disorder of the pancreas, and is a potentially life-threatening disease. The clinical presentation of acute pancreatitis ranges from mild, which is found in 70–80% of cases, to severe, which is associated with a high mortality rate from AP-related complications [1, 2]. The etiologies and pathogenesis of AP have been the subjects of continuous research. Various studies suggest that the pathology of AP arises from a premature activation of intra-acinar enzymes leading to autodigestion of pancreas. This promotes the synthesis and release of many pro-inflammatory cytokines and chemokines and the induction of oxidative stress leading to local inflammation [1–3]. AP is characterized by interstitial edema, acinar cell necrosis, hemorrhages, and neutrophil infiltration. Moreover, the release of pro-inflammatory mediators into the circulation triggers the development of systemic inflammation, resulting in multiple organ dysfunction syndrome [4, 5]. Over the past three decades, despite the tremendous effort to find the specific treatment for AP, the standard of care for patients with AP remains supportive. This lack of targeted therapy is mainly due to our incomplete understanding of the underlying mechanism of AP [2].

Phytoestrogens are natural chemical compounds derived from plants, which have structures and functions similar to endogenous estrogens. Genistein (4’, 5, 7—trihydroxylisoflavone) (GEN) is a phytoestrogen that belongs to the category of isoflavones. The pharmacological activity of genistein is usually via estrogen receptors due to its similar structure to estradiol [6]. In addition, genistein has been extensively used as an antioxidant agent. The direct antioxidant property of genistein derives from its structure as it is able to donate hydrogen from phenolic hydroxyl groups to free radical molecules, thus acting as a free radical scavenger [7, 8]. Genistein can also increase the activity of antioxidant enzymes, including superoxide dismutase, glutathione reductase, and glutathione peroxidase in 12-O-tetradecanoylphorbol-13-acetate (TPA) -induced H_2_O_2_ formation and superoxide anion (O_2_^−^) generation by xanthine/xanthine oxidase in HL-60 cells and the mouse skin tumorigenesis model [9]. Furthermore, genistein also possesses anti-inflammatory properties. Genistein has been shown to reduce c-reactive protein (CRP), tumor necrosis factor (TNF- α), and transforming growth factor beta-1 (TGF-β1) in streptozotocin induced diabetic rats [10]. In addition, genistein also reduced interleukin 1 beta (IL-1β), TNF-α, interleukin-6 (IL-6) and nuclear factor kappa B (NF-kB) production in lipopolysaccharide (LPS)-treated RAW 264.7 macrophages [11, 12]. Hence, the aim of this study was to determine the effects of genistein on oxidative stress and inflammatory markers in AP mice.

## Materials and methods

### Animals

Four-week-old male ICR mice weighing 30–40 g were purchased from the Nation Laboratory Animal Center, Salaya Campus, Mahidol University, Nakhon Pathom, Thailand. The experimental protocol was approved by the Ethical Committee, Faculty of Medicine, Chulalongkorn University, Bangkok, Thailand (No.12/2559). All experiments were conducted in accordance with the Ethical Principles and Guidelines for the Use of Animals by the National Research Council of Thailand. Mice were acclimatized at least 1 week before the experiment in a controlled temperature room at 25 ± 1 ºC with 12:12 h light–dark cycle and were fed with standard diet ad libitum.

Since this was the first study to evaluate the therapeutic effect of genistein in AP, we used the amylase levels from a study by Siriviriyakul et al. [13] to calculate the sample size using the program G Power version 3.1.9.7 at the alpha level of 0.05 and the power of 0.90. The calculated sample size in each group was 6 (Suppl Fig. 1). The aforementioned study evaluated the effects of low- and high-dose curcumin in a mouse model of AP.

### Experimental protocols

Twenty-four male ICR mice were equally divided into 4 groups. In the control group (Con group), a once-daily intraperitoneal (IP) injection of 2% DMSO was given to each mouse for 4 days. In the acute pancreatitis group (AP group), two doses of 350 mg/100 g body weight of L-arginine or L-arg (Sigma-Aldrich Inc, St. Louis, MO, USA) dissolved in 0.9% normal saline at pH 7.0 were injected intraperitoneally 1 h apart. In the AP and low-dose genistein group (LG group), 10 mg/kg genistein (Sigma-Aldrich Inc, St. Louis, MO, USA) in 2% DMSO was given by IP injection 2 h prior to L-arg injection, followed by once-daily injection of genistein for 3 days. In the AP and high-dose genistein group (HG group), 100 mg/kg genistein in 2% DMSO was given by IP injection 2 h prior to L-arg injection, followed by once-daily dosing for 3 days. The genistein dose was modified from the dosage used in a murine model of non-alcoholic fatty liver disease [14].

All mice were euthanized by intraperitoneal injection of sodium thiopental (50 mg/kg body weight) 72 h after L-Arg injection. Pancreas tissue was obtained and divided into 2 parts. The first part was formalin-fixed for histopathological examination and for immunohistochemical detection of myeloperoxidase (MPO), NF-kB, 4-hydroxynonenal (4-HNE), and apoptosis. The second part was stored at -80 °C until the time of analysis for malondialdehyde (MDA). Cardiac puncture was performed to obtain blood samples. The sample was stored at 25 °C for 2 h until the blood has clotted. The clotted sample was then centrifuged for 20 min at the speed of 1000 × g with the set temperature of 4 °C. Serum samples were kept at -80 °C until further analysis (amylase, IL-6, and CRP).

### Body weight change

All animals were weighed weekly. Total body weight change (in gram) was defined by the difference in body weight at the beginning and at the end of the study in each mouse.

### Biochemical analysis of serum amylase (AMY)

Amylase activity was measured via the colorimetric method using the biochemical analyzer Reflotron®Plus (Lot no. 11200658202, Roche Diagnostics, Rotkreuz, Switzerland). Serum was pipetted onto a reagent strip designed for the quantitative determination of amylase level, which was then inserted in an automated machine. The results were expressed as enzyme concentration (unit/liter).

### The determination of serum interleukin-6 and c-reactive protein levels

Serum IL-6 and CRP levels were measured using commercial enzyme-linked immunosorbent assay (ELISA) kits for mouse IL-6 and CRP (Lot no. M6000B for IL-6 and MCRP00 for CRP, R&D system, Inc., USA). The procedures were performed according to manufacturer’s instruction. IL-6 and CRP levels in serum samples were determined using the standard curve of each assay and were expressed as pg/mL and ng/mL, respectively.

### Pancreatic malondialdehyde determination

MDA level was measured from the homogenized pancreatic tissue using a commercial assay kit (Lot no. CAY-10009055, Cayman Chemical Company, USA). The principle of the assay is to measure the rate of production of thiobarbituric acid-reactive under high temperature and acidic conditions. First, pancreas tissue was homogenized in RIPA buffer with protease inhibitor on ice by a sonicate machine for 15 s. Then, the pancreas tissue homogenates were centrifuged at 1600 × g for 10 min at 4˚C and the supernatants were collected. Following the manufacturer’s protocol, the absorbance of the supernatant fraction was determined at a wavelength of 532 nm and MDA levels were calculated from a standard curve which were expressed as nmol/mg protein.

### Immunohistochemistry for the determination of myeloperoxidase activity, nuclear factor-kappa beta and 4-hydroxynonenal expressions

Paraffin-embedded samples were cut into 4-μm sections and then deparaffinized with ethanol. Antigen retrieval was achieved by incubating samples with appropriate buffers (EDTA at pH 8.0 for MPO, citrate buffer at pH 6.0 for NF-kβ and 4-HNE) in a microwave for 13 min. Endogenous peroxidase activity and non-specific binding were blocked using 3% hydrogen peroxide and 3% normal horse serum, respectively. Samples were then washed with phosphate-buffered saline (PBS) at pH 7.4 for 5 min. Subsequently, slides were incubated with an antibody against MPO (Lot no. AF3667, Dako, Denmark; at a dilution of 1:500), a polyclonal antibody against the p65 subunit of NF-kB (Lot no. 8242S, Cell Signaling Technology, Inc., USA; at a dilution of 1:400), and 4-HNE (Lot no. MAB3249, R&D Systems, Inc., USA; at a dilution of 1:500) at 4 °C overnight to determine the MPO activity, the expression of NF-kB, and 4-HNE, respectively. Then tissues were incubated with secondary antibody for each assay (Dako, Denmark for MPO and Abcam, MA, USA for NF-kB and 4-HNE) for 30 min at 25 °C. After the development of color with diaminobenzidine (DAB), slides were counterstained with hematoxylin. Under a light microscope, positive cells were defined as pancreatic acinar cells with dark brown nuclei. The numbers of positive cells were counted by the Aperio ImageScope software (Leica Biosystems Imaging, Inc., MD, USA) in 10 randomly selected fields at a magnification of 400. The results were expressed as the average number of positive-stained cells per high-power field (HPF) for MPO activity, and as a percentage of positive cells for NF-kB and 4-HNE expressions.

### The determination of acinar cell apoptosis

We determined the degree of pancreatic acinar cell apoptosis using the terminal deoxynucleotidyl transferase biotin-dUTP nick end labeling (TUNEL) method (Lot no. CMI-3S7101); the performance of which followed the manufacturer's manual. Acinar cells with dark brown nuclei were counted as TUNEL positive cells using the Aperio ImageScope software (Leica Biosystems Imaging, Inc., MD, USA). A percentage of TUNEL positive cells represented the degree of apoptotic acinar cells in each group.

### Pathological examination of pancreas

An experienced pathologist who was blinded to the experiment reviewed histological slides and graded the severity of acute pancreatitis according to the scoring system suggested by H. E. V. DE COCK [15]. In brief, three lesions (neutrophilic inflammation, interstitial edema, and mesenteric fat necrosis) were each given a point based on their severity. The points for each lesion were summed with a maximal score of 9. The histological grading was classified as normal (score = 0), mild AP (score = 1–3), moderate AP (score = 4–6), and severe AP (score = 7–9).

### Statistical analyses

All data were presented as mean and standard error of mean (SEM). For comparison among groups of animals, one-way analysis of variance (one-way ANOVA) with LSD’s post-hoc comparisons test was used. Descriptive statistics was used for the histological examination of the pancreas. Differences were considered statistically significant at *p* value < 0.05.

## Results

### The effect of genistein on body weight change

**Fig. 1:**
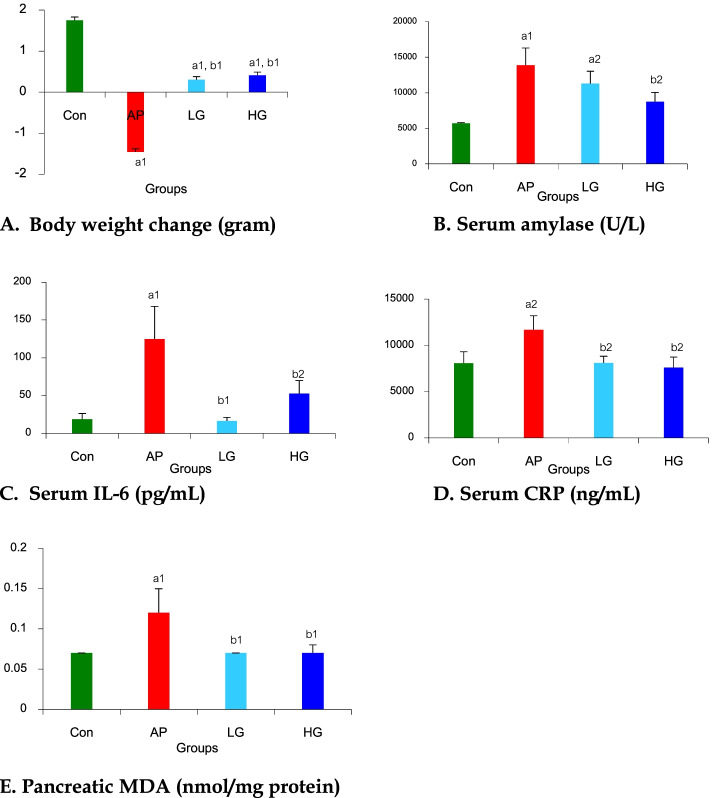
Effects of genistein on **A** body weight change, **B** serum amylase, **C** serum IL-6, **D** serum CRP, and **E** pancreatic MDA in mice with acute pancreatitis. Data are expressed as mean ± SEM. ^a1^*p* < 0.01, ^a2^*p* < 0.05 *vs.* Con group; ^b1^*p* < 0.01, ^b2^*p* < 0.05 *vs.* AP group (*n* = 6 mice per group). Con, Control group; AP, Acute pancreatitis group; LG, Low-dose genistein group; HG, High-dose genistein group

The result of weight change was shown in Fig. 1A. Body weight increased in the control group (∆body weight, 1.75 ± 0.08 g) but significantly decreased in the AP group when compared with the Con group (∆body weight, -1.46 ± 0.15 *vs.* 1.75 ± 0.08 g, respectively, *p* < 0.01). Body weight significantly increased in LG (∆body weight, 0.30 ± 0.27 *vs.* -1.46 ± 0.15 g, respectively, *p* < 0.01) and HG (∆body weight, 0.41 ± 0.22 *vs.* -1.46 ± 0.15 g, respectively, *p* < 0.01) groups when compared with the AP group. However, there were no differences in body weight changes between LG and HG groups.

### The effect of genistein on serum amylase level

The result of serum AMY was showed in Fig. 1B. The significant increase in serum AMY levels was seen in the AP group when compared with the Con group (13,860.00 ± 2,416.12 *vs.* 5,714.00 ± 82.10 U/L, respectively, *p* < 0.01). A non-significant decrease in serum AMY was noted in the LG group when compared with AP group (11,283.67 ± 1,725.48 *vs.* 13,860.00 ± 2,416.12 U/L, respectively), whereas a significant decline in serum AMY was seen in the HG group when compared with the AP group (8,728.33 ± 1,311.95 *vs.* 13,860.00 ± 2,416.12 U/L, respectively, *p* < 0.05). However, there were no differences in serum AMY levels between LG and HG groups.

### The effect of genistein on serum IL-6 and serum CRP levels

Serum IL-6 levels in all groups were shown in Fig. 1C. Serum IL-6 levels significantly increased in the AP group when compared with the Con Group (124.68 ± 43.39 *vs.* 18.59 ± 7.72 pg/mL, respectively, *p* < 0.01) and significantly decreased in LG (16.61 ± 4.59 *vs.* 124.68 ± 43.39 pg/mL, respectively, *p* < 0.01) and HG (52.58 ± 17.43 *vs.* 124.68 ± 43.39 pg/mL, respectively, *p* < 0.05) groups when compared with the AP group. No differences in serum IL-6 levels were seen between LG and HG groups.

Serum CRP levels in all groups were shown in Fig. 1D. Serum CRP levels significantly increased in the AP group when compared with the Con Group (11,687.07 ± 1,507.23 *vs.* 8,068.63 ± 1,251.38 ng/mL, respectively, *p* < 0.05) and significantly decreased in LG (8,094.60 ± 732.82 *vs.* 11,687.07 ± 1,507.23 ng/mL, respectively, *p* < 0.05) and HG (7,607.77 ± 1,125.92 *vs.* 11,687.07 ± 1,507.23 ng/mL, respectively, *p* < 0.05) groups when compared with the AP group. No differences in serum CRP levels were seen between LG and HG groups.

### The effect of genistein on pancreatic MDA level

Results of pancreatic MDA in all groups were shown in Fig. 1E. Pancreatic MDA levels significantly increased in the AP group when compared with the Con Group (0.12 ± 0.03 *vs.* 0.07 ± 0.00 nmol/mg protein, respectively, *p* < 0.01) and significantly decreased in LG (0.07 ± 0.00 *vs.* 0.12 ± 0.03 nmol/mg protein, respectively, *p* < 0.01) and HG (0.07 ± 0.01 *vs.* 0.12 ± 0.03 nmol/mg protein, respectively, *p* < 0.01) groups when compared with the AP group. There were no differences in pancreatic MDA levels between LG and HG groups.

### The effect of genistein on histopathology and apoptosis (TUNEL)

**Fig. 2:**
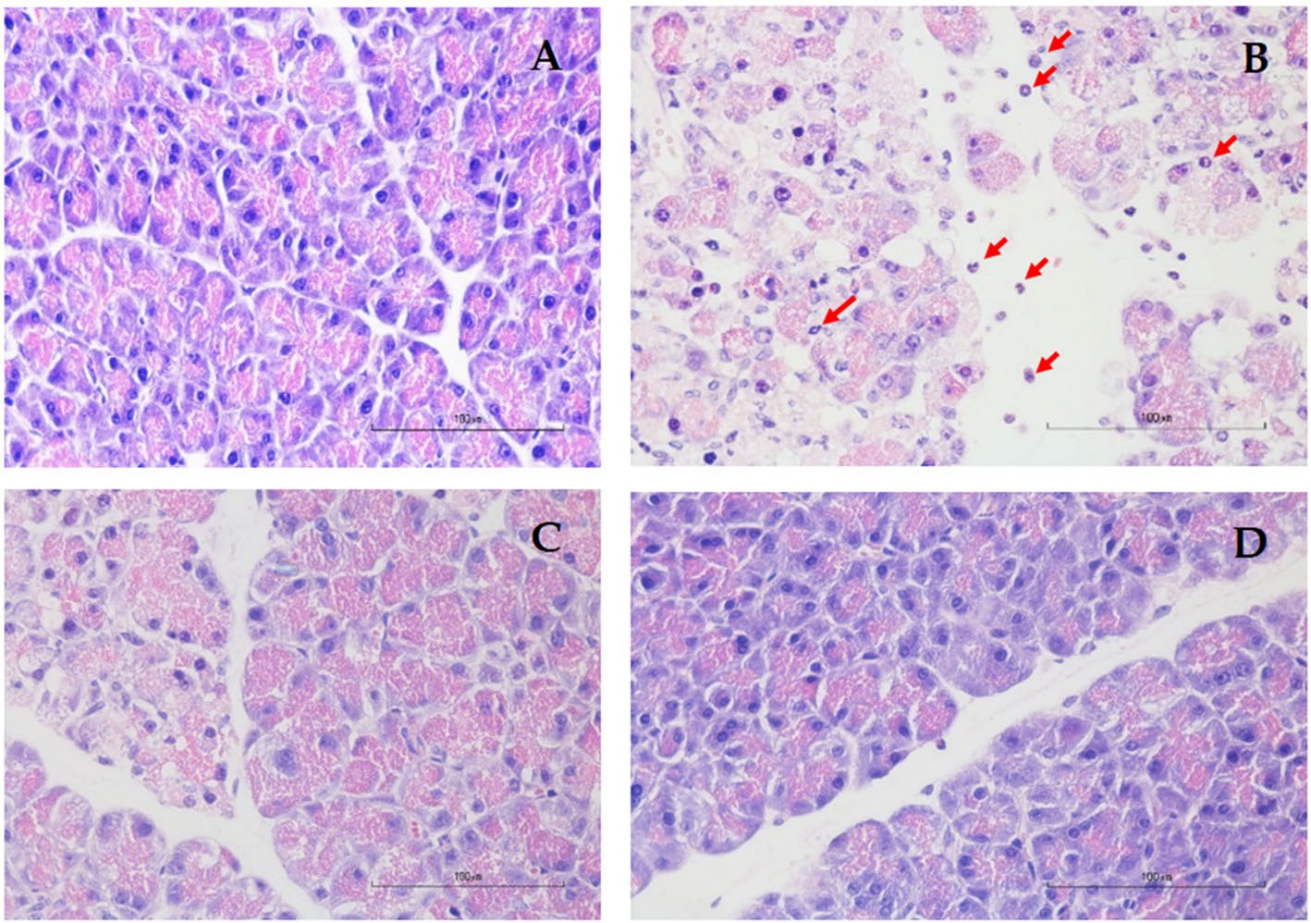
Pancreatic histopathology of hematoxylin–eosin staining at a magnification of 400, scale bar 100 µm. **A** Control group showed normal pancreatic architecture without any evidence of tissue damage; **B** Acute pancreatitis group showed severe tissue damage characterized by neutrophil infiltration (red arrow), edema, and acinar cell necrosis; **C** Low-dose genistein group showed reduced neutrophil infiltration, edema, and acinar cell necrosis; **D** High-dose genistein group showed reduced neutrophil infiltration, edema, and acinar cell necrosis

Detailed histopathological scores of each component and the total severity score of each group were shown in Table 1. As shown in Fig. 2, there was evidence of pancreatic injury/inflammation in the AP group. The administration of L-arginine induced extensive tissue damage characterized by neutrophil infiltration, edema, and acinar cell necrosis. After treatment with low- and high-dose genistein, the damage was mild as shown by significantly decreased histopathological scores in genistein groups when compared with the AP group. No differences in histopathological scores were seen between LG and HG groups.

**Table 1:** Histopathology scores and severity of acute pancreatitis in all groups

Groups	*n*	Pancreas pathology												Severity			
		**Neutrophil infiltration**				**Edema**				**Necrosis**				**None**	**Mild**	**Moderate**	**Severe**
		0	1	2	3	0	1	2	3	0	1	2	3				
Con	6	6		-	-	6	-	-	-	6	-	-	-	6	-	-	-
AP	6	-	3	1	2	-	2	3	1	-	2	3	1	-	-	4	2
LG	6	1	4	1	-	-	4	2	-	1	3	2	-	-	4	2	-
HG	6	5	-	1	-	-	6	-	-	3	2	-	1	-	5	1	-

Each section was scored according to the criteria described by H. E. V. DE COCK et al. in 2007 [15]. The histological grading ranges from 0–3 (0 = Not present; 1 = Mild or < 25% of the pancreatic parenchyma; 2 = Moderate or present in 25–50% of the parenchyma; 3 = Severe or > 50% of the parenchyma)

The severity of AP was classified according to the total score as follows: 0 = none 1–3 = mild 4–6 = moderate 7–9 = severe

*Abbreviations*: *Con* Control group, *AP* Acute pancreatitis group, *LG* Low-dose genistein group, *HG* High-dose genistein group

**Fig. 3:**
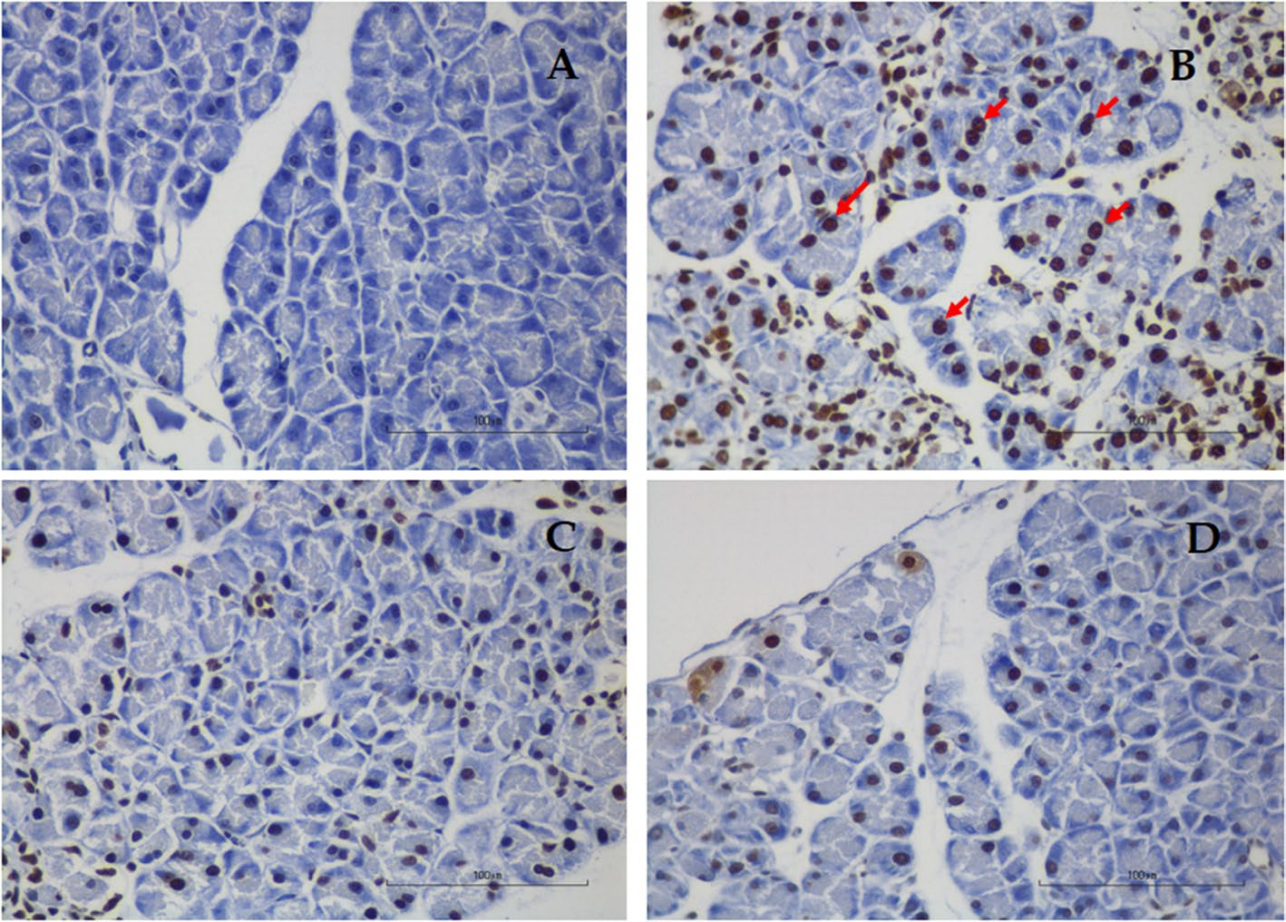
Representative images of TUNEL staining for the evaluation of apoptotic acinar cells at a magnification of 400, scale bar 100 µm. **A** Control group; **B** Acute pancreatitis group; **C** Low-dose genistein group; **D** High-dose genistein group. Red arrows indicate positive TUNEL staining cells

TUNEL staining in each group was shown in Fig. 3 and Table 2. Apoptotic acinar cells are those with dark brown stain. The percentage of apoptotic acinar cells in the AP group was significantly higher than in the Con group (0.22 ± 0.01 *vs.* 0.07 ± 0.01%, respectively, *p* < 0.01). The percentage of apoptotic acinar cells was significantly reduced in LG (0.09 ± 0.00 *vs*. 0.22 ± 0.01%, respectively, *p* < 0.01) and HG (0.07 ± 0.00 *vs.* 0.22 ± 0.01%, respectively, *p* < 0.01) groups when compared with the AP group. The degree of apoptosis was not different between LG and HG groups.

**Table 2:** Effect of genistein on MPO, NF-kB, 4-HNE expression and acinar cell apoptosis in acute pancreatitis mice

Groups (*n* = 6)	Parameters			
	**MPO positive (cells of HPF)**	**NF-kB Positive cells (%)**	**4-HNE Positive cells (%)**	**TUNEL Positive cells (%)**
Con	0.33 ± 0.08	0.44 ± 0.01	0.40 ± 0.02	0.07 ± 0.01
AP	21.00 ± 4.60^a1^	0.75 ± 0.01^a1^	0.76 ± 0.01^a1^	0.22 ± 0.01^a1^
LG	4.13 ± 0.73^b1^	0.47 ± 0.01^a2,b1^	0.45 ± 0.01^a2,b1^	0.09 ± 0.00^a2,b1^
HG	2.97 ± 0.44^b1^	0.46 ± 0.01^b1^	0.43 ± 0.01^b1^	0.07 ± 0.00^b1^

Data are express as mean ± SEM. ^a1^*p* < 0.01, ^a2^*p* < 0.05 *vs.* Con group; ^b1^*p* < 0.01, ^b2^*p* < 0.05 *vs.* AP group

*Abbreviations*: *Con* Control group, *AP* Acute pancreatitis group, *LG* Low dose of genistein group, *HG* High dose of genistein group, *MPO* Myeloperoxidase, *NF-kB* Nuclear factor-kappa beta, *4-HNE* 4-Hydroxynonenal, *TUNEL* Terminal deoxynucleotidyl transferase dUTP nick end labeling

### The effect of genistein on MPO positive cells

**Fig. 4:**
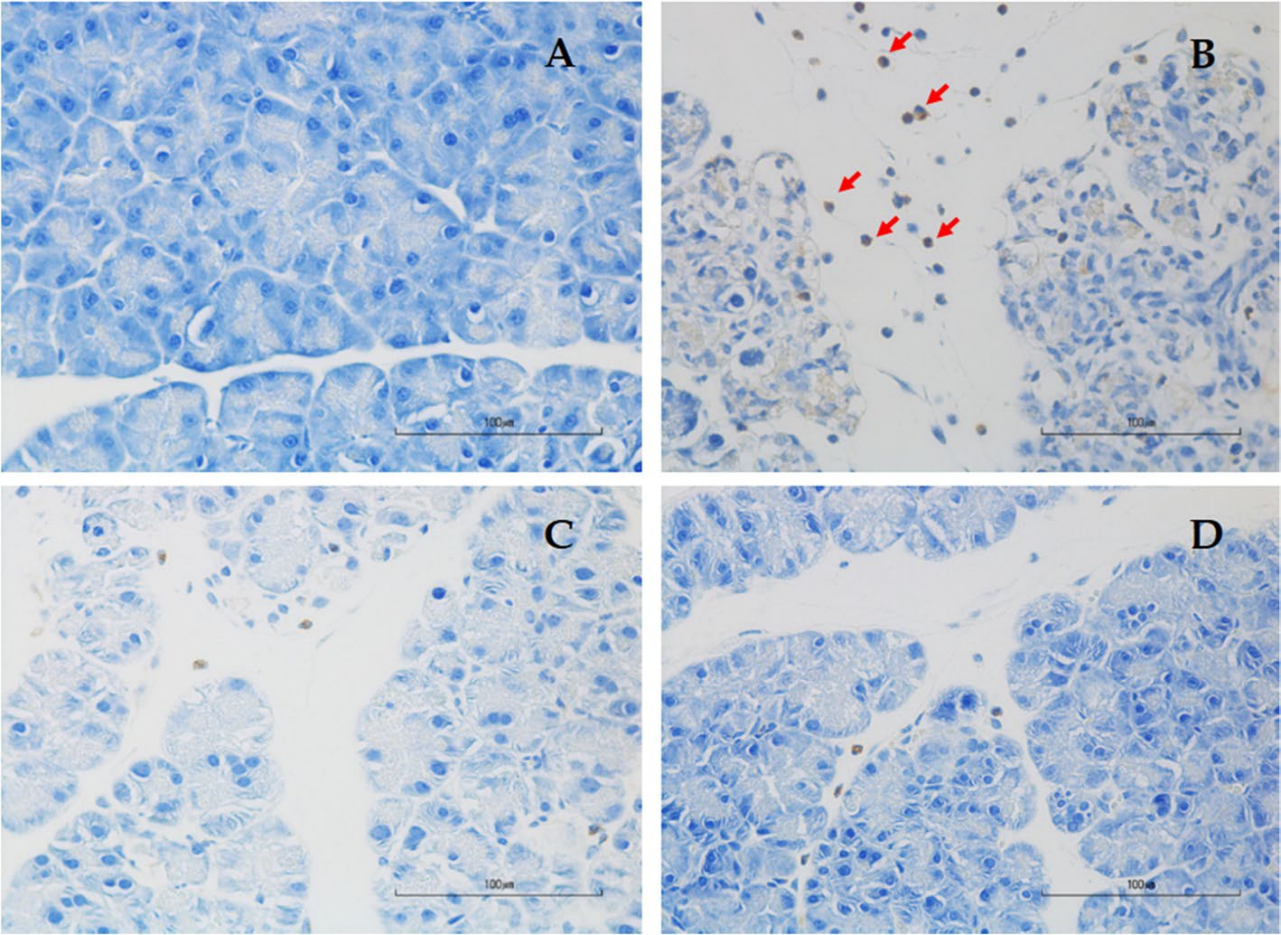
Representative images of immunohistochemistry for MPO expression in the pancreas at a magnification of 400, scale bar 100 µm. **A** Control group; **B** Acute pancreatitis group; **C** Low-dose genistein group; **D** High-dose genistein group. Red arrows indicate positive MPO staining cells

Results of MPO positivity was shown in Fig. 4 and Table 2. The number of MPO positive cells infiltrating in the pancreas was markedly increased in the AP group when compared with the Con group (21.00 ± 4.60 *vs.* 0.33 ± 0.08 in 10 fields at a magnification of 400, respectively, *p* < 0.01). Comparing with the AP group, MPO positivity significantly reduced in the LG group (21.00 ± 4.60 *vs.* 4.13 ± 0.73 in 10 fields at a magnification of 400, respectively, *p* < 0.01) and HG group (21.00 ± 4.60 *vs.* 2.97 ± 0.44 in 10 field at a magnification of 400, respectively, *p* < 0.01) groups. However, there was no difference in MPO positivity between LG and HG groups.

### Effects of genistein on NF-kB expression

**Fig. 5:**
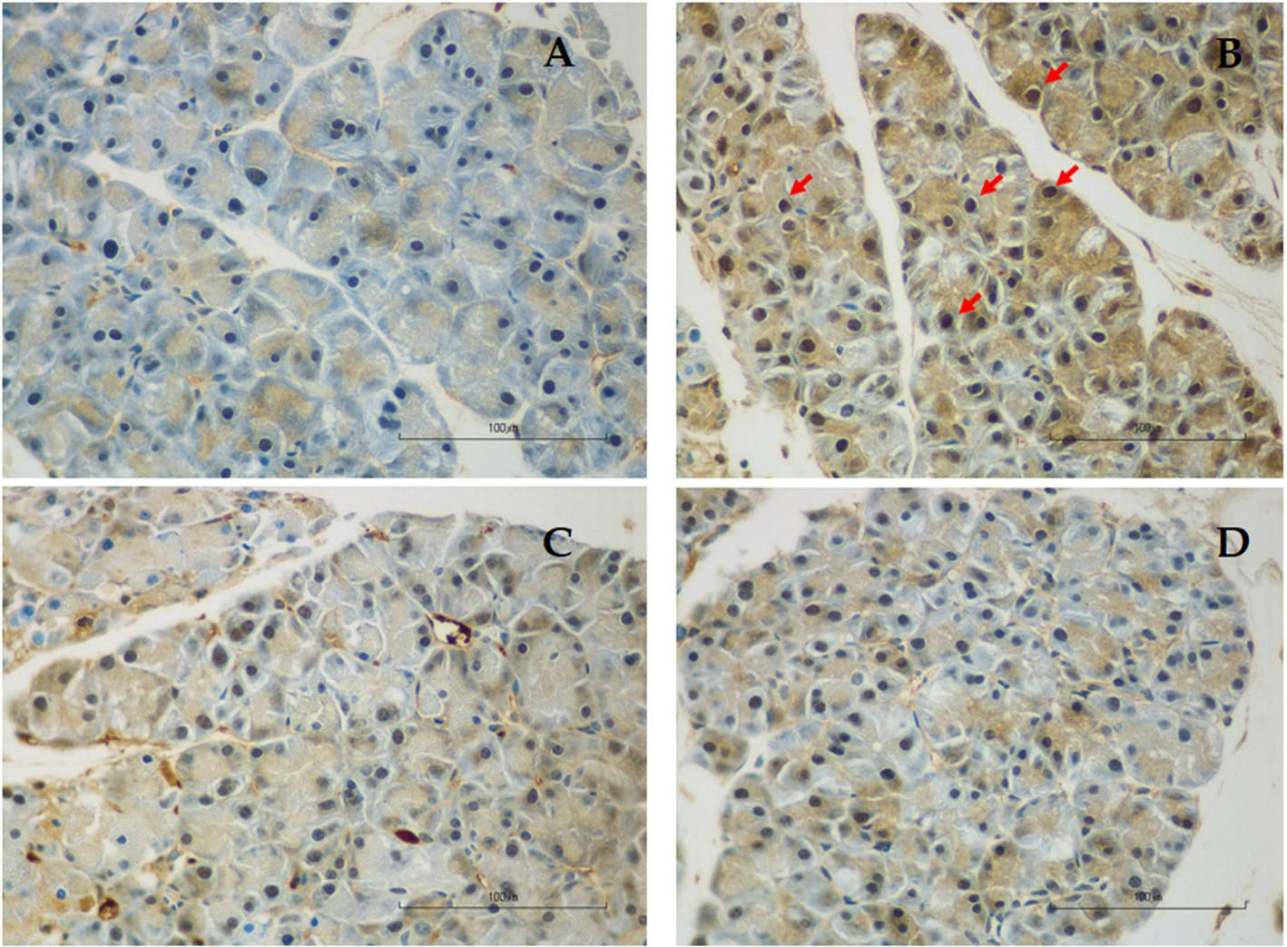
Representative images of immunohistochemistry for NF-kB expression in the pancreas at a magnification of 400, scale bar 100 µm. **A** Control group; **B** Acute pancreatitis group; **C** Low-dose genistein group; **D** High-dose genistein group. Red arrows indicate positive NF-kB staining cells

Results of NF-kB positivity were shown in Fig. 5 and Table 2. The percentage of NF-kB-positive cells in the AP group was significantly higher than in the Con group (0.75 ± 0.01 *vs.* 0.44 ± 0.01, respectively, *p* < 0.001). The percentage of NF-kB-positive cells significantly declined in LG (0.47 ± 0.01 *vs.* 0.75 ± 0.01%, respectively, *p* < 0.001) and HG (0.46 ± 0.01 *vs.* 0.75 ± 0.01%, respectively, *p* < 0.001) groups as compared with the AP group. There was no difference in NF-kB positivity between LG and HG groups.

### Effects of genistein on 4-HNE expressions

**Fig. 6:**
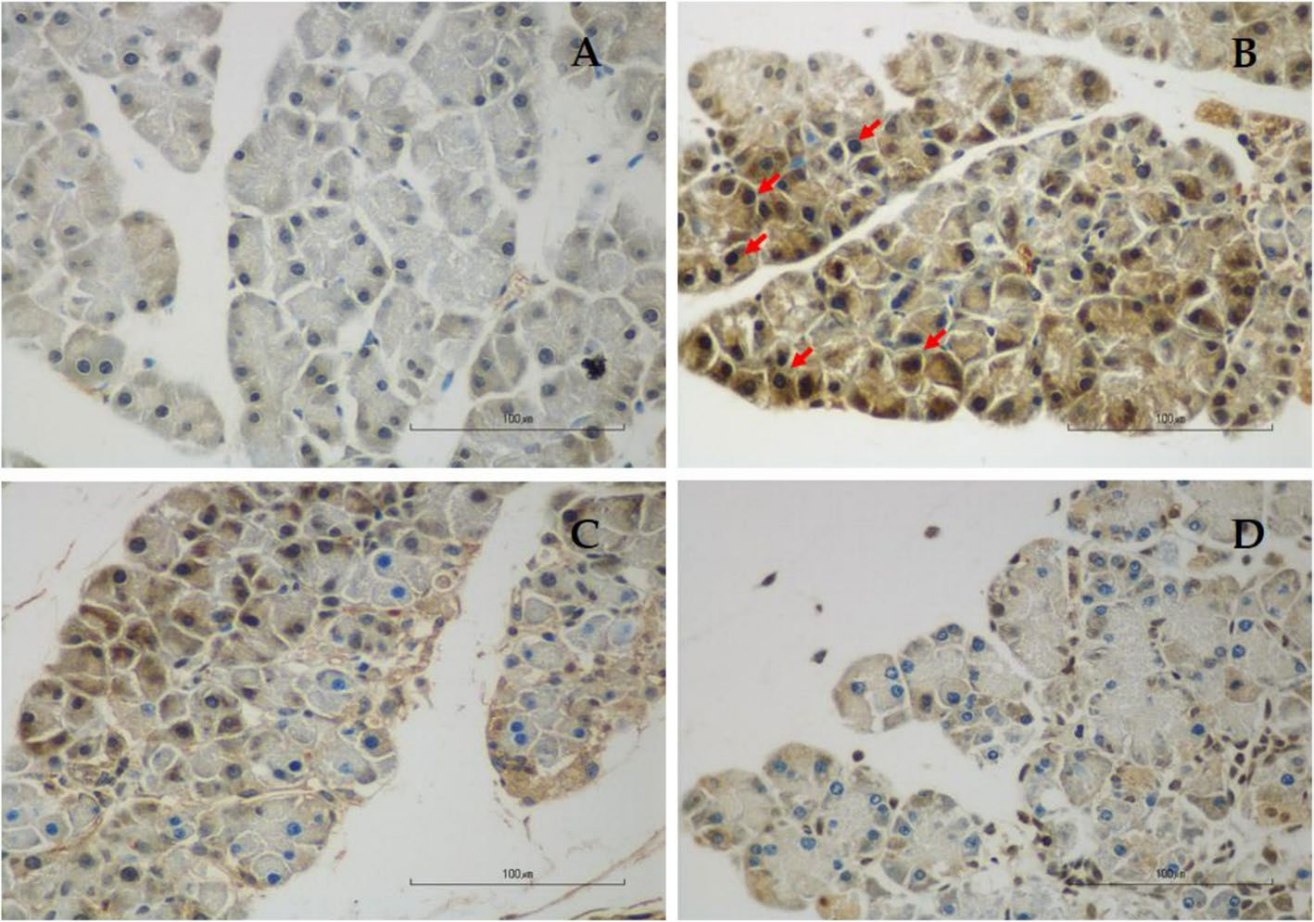
Representative images of immunohistochemistry for 4-HNE expression in the pancreas at a magnification of 400, scale bar 100 µm. **A** Control group; **B** Acute pancreatitis group; **C** Low-dose genistein group; **D** High-dose genistein group. Red arrows indicate positive 4-HNE staining cells

Results of 4-HNE positivity were shown in Figs. 6 and Table 2. The percentage of 4-HNE-positive cells in the AP group was significantly higher than in the Con group (0.76 ± 0.01% *vs.* 0.40 ± 0.02%, respectively, *p* < 0.001). The percentage of 4-HNE-positive cells significantly declined in LG (0.45 ± 0.01% *vs.* 0.76 ± 0.01%, respectively, *p* < 0.001) and HG (0.43 ± 0.01% *vs.* 0.76 ± 0.01%, respectively, *p* < 0.001) groups as compared with the AP group. No difference in 4-HNE positivity was seen between LG and HG groups.

## Discussion

The present study was the first to report that genistein could alleviate the severity of acute pancreatitis. The administration of L-arginine in this study could successfully induce acute pancreatitis as evidenced by body weight loss and increases in serum amylase, serum IL-6, serum CRP, oxidative stress markers (pancreatic MDA and 4-HNE), percentages of MPO positive cells and NF-kB positive cells, pancreatic apoptosis, and pancreatic histopathological changes.

Genistein (4’, 5, 7—trihydroxylisoflavone) is a phytoestrogen that belongs to the category of isoflavones. In addition to its estrogenic activity [6], genistein acts both directly and indirectly as an antioxidant agent. Studies have shown beneficial effects of genistein on oxidative stress and inflammation in various conditions [16–24]. In this study, we demonstrated that genistein could counteract the effects of L-arginine on both pancreatic histology and laboratory parameters. Injection of genistein in both low and high does reduced serum AMY, inflammatory markers (IL-6 and CRP), pancreatic MDA, expressions of MPO and NF-kB, pancreatic apoptosis, and prevented pancreatic histological damage. Nonetheless, a dose dependent effect of genistein was not seen in this study.

Serum amylase is the important marker of acute pancreatitis as they are secreted by acinar cells of the pancreas. In accordance with previous studies, in the present study, L-arginine administration led to significantly increased serum AMY at the end of the study, which was corresponding with histopathologic changes of acute pancreatitis [25]. Genistein treatment significantly decreased amylase levels, more prominently in the high-dose group, when compared with the non-treatment group. In agreement with serum amylase, genistein ameliorated the degree of neutrophil infiltration, edema, and necrosis on pancreatic histopathology.

Interleukin-6 is a principle mediator which has a role in the regulation of immune response and inflammatory process [26, 27]. In addition, CRP, an acute phase reactant synthesized by the liver, is usually elevated in inflammatory conditions and has been shown to be an accurate severity predictor [28]. Recent studies demonstrated the elevation of IL-6 and CRP in animal models of caeruline [29] and L-arginine induced acute pancreatitis [30]. In agreement with other studies, serum IL-6 and serum CRP levels increased in L-arginine-treated mice in the current study. Treatment with genistein in both low and high doses effectively decreased levels of serum IL-6 and serum CRP suggesting the anti-inflammatory effect of genistein in acute pancreatitis. Genistein has previously been shown to reduce IL-6 production through the inhibition of extracellular signal-regulated kinase (ERK) mitogen-activated protein kinase (MAPK) pathways [31].

Neutrophil sequestration in inflamed tissues can be quantified by measuring the tissue MPO activity. MPO has been implicated in the promotion of tissue damage in various inflammatory diseases [32]. Our results demonstrated that genisein in both low and high doses reduced MPO activity and neutrophil infiltration**.** In a model of acute lung injury, genistein has been shown to suppress cytokine-inducible neutrophil chemoattractant (CINC) and matrix metalloproteinase-9 (MMP-9) production leading to the inhibition of neutrophil infiltration and activation, and the reduction of MPO activity [33, 34].

Malondialdehyde is an indicator of oxidative stress in cell and tissue and is formed as a result of lipid peroxidation of polyunsaturated fatty acids [34]. Previous studies showed that MDA levels were elevated in animal model of L-arginine or caerulein induced pancreatitis suggesting the role of oxidative stress in the pathogenesis of acute pancreatitis [35, 36]. Likewise, Lipid peroxidation of polyunsaturated fatty acids also produces 4-HNE, another oxidative stress marker. In this study, 4-HNE expression increased in mice with acute pancreatitis and reduced after genistein treatment in both low- and high-dose groups. Due to the ability of genistein to scavenge free oxygen radicals [8, 37, 38], it was not surprising that both low- and high-dose genistein could reduce oxidative stress as indicated by suppressed tissue MDA levels and 4-HNE expression, and thus mitigating lipid peroxidation and cell membrane damage in mice with acute pancreatitis in this study.

Nuclear factor-kappa beta activation is a key event in the development of acute pancreatitis. Pathologic calcium signaling and ROS generation have been shown to be important mediators in the activation of NF-kB [39]. A study using transgenic mice demonstrated that the higher level of NF-kB activity was associated with the increased severity of acute pancreatitis, and persistent elevation of NF-kB levels could lead to pathological changes seen in chronic pancreatitis [40]. In this study, we found the increased NF-kB expression in mice with acute pancreatitis, which was attenuated by the treatment with both low and high-dose genistein. Genistein has been shown to block the degradation of IκB-α, thus inhibiting the nuclear translocation and subsequent activation of NF-kB in LPS-activated BV2 microglia [17].

An in vitro study has shown that L-Arginine could modulate the expression of pancreatitis-associated protein (PAP) gene, which led to acinar cell apoptosis [41]. Moreover, products of lipid peroxidation, such as 4-HNE, could induce the Fas expression, and the phosphorylation and nuclear translocation of p53, which subsequently activates caspase 3 and acinar cell apoptosis [42]. In the current study, the degree of acinar cell apoptosis was significantly higher in the L-Arginine induced AP group than in the control group. Both low and high doses of genistein reduced the number of apoptotic acinar cells to the level observed in the control group. Genistein likely alleviated the degree of acinar cell apoptosis through its antioxidant effect.

## Conclusion

In conclusion, genistein treatment in both low and high doses attenuated the severity of acute pancreatitis via the reduction of inflammation, oxidative stress, and apoptosis in this mouse model of L-arginine induced acute pancreatitis. Clinical studies are warranted to confirm the efficacy of genistein in the treatment of patients with acute pancreatitis. We also have to take into the consideration the oral bioavailability of genistein in human and the application of drug delivery system for the successful outcomes in clinical trials [43].

## Supplementary Information

**Additional file 1: Supplementary figure 1.** Sample size calculation using G Power analysis. Sample size was calculated using serum amylase levels in the control and low-dose curcumin groups in a study by Siriviriyakul *et al*.^13^. With the alpha of 0.05 and the power of 90%, the sample size in each group was 6.

## Data Availability

The datasets used and/or analyzed during the current study are available from the corresponding author on reasonable request due to privacy reasons.
