# Supplementary material for: Genistein attenuated oxidative stress, inflammation, and apoptosis in L-arginine induced acute pancreatitis in mice
Source: BMC Complement Med Ther. 2022 Aug 4;22:208. doi: 10.1186/s12906-022-03689-9 (PMC9351145; doi:10.1186/s12906-022-03689-9)
Supplement: Supplementary file 1 — Additional file 1: Supplementary figure 1. Sample size calculation using G Power analysis. Sample size was calculated using serum amylase levels in the control and low-dose curcumin groups in a study by Siriviriyakul et al.13. With the alpha of 0.05 and the power of 90%, the sample size in each group was 6. [file 12906_2022_3689_MOESM1_ESM.docx]

**Supplementary figure 1**. Sample size calculation using G Power analysis


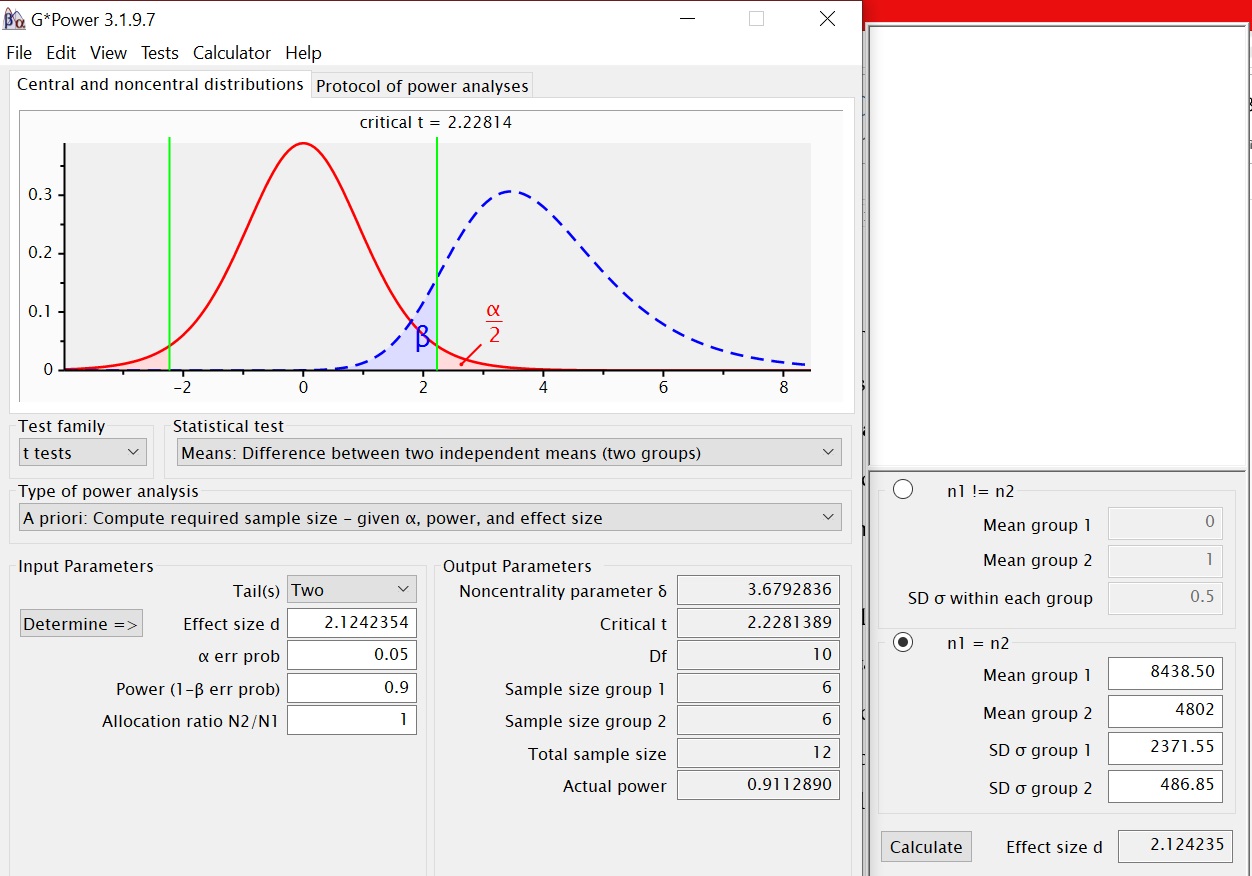


Sample size was calculated using serum amylase levels in the control and low-dose curcumin groups in a study by Siriviriyakul *et al*.^13^. With the alpha of 0.05 and the power of 90%, the sample size in each group was 6.
